# Spatial transcriptomics: recent developments and insights in respiratory research

**DOI:** 10.1186/s40779-023-00471-x

**Published:** 2023-08-17

**Authors:** Wen-Jia Wang, Liu-Xi Chu, Li-Yong He, Ming-Jing Zhang, Kai-Tong Dang, Chen Gao, Qin-Yu Ge, Zhou-Guang Wang, Xiang-Wei Zhao

**Affiliations:** 1 State Key Laboratory of Bioelectronics, School of Biological Science and Medical Engineering, Southeast University, Nanjing, 210096 China; 2 Oujiang Laboratory (Zhejiang Lab for Regenerative Medicine, Vision and Brain Health), School of Pharmaceutical Sciences, Wenzhou Medical University, Wenzhou, 325035 Zhejiang China; 3 Orthopaedic Bioengineering Research Group, Division of Surgery and Interventional Science, University College London, London, HA7 4LP UK

**Keywords:** Spatial transcriptomics, Lung, Tumor, Spatial multi-omics

## Abstract

The respiratory system’s complex cellular heterogeneity presents unique challenges to researchers in this field. Although bulk RNA sequencing and single-cell RNA sequencing (scRNA-seq) have provided insights into cell types and heterogeneity in the respiratory system, the relevant specific spatial localization and cellular interactions have not been clearly elucidated. Spatial transcriptomics (ST) has filled this gap and has been widely used in respiratory studies. This review focuses on the latest iterative technology of ST in recent years, summarizing how ST can be applied to the physiological and pathological processes of the respiratory system, with emphasis on the lungs. Finally, the current challenges and potential development directions are proposed, including high-throughput full-length transcriptome, integration of multi-omics, temporal and spatial omics, bioinformatics analysis, etc. These viewpoints are expected to advance the study of systematic mechanisms, including respiratory studies.

## Background

The respiratory system comprises the respiratory tract and lungs and is one of the organs that directly interface with the external environment [1]. The complexity of its structure and function hinders our understanding of the physiological and pathological processes involved [2]. The pathogenesis of most common respiratory diseases is complicated, a significant public health problem. Lung cancer is one of the most common diseases in clinical practice [3], with its morbidity and mortality ranking first among all tumor types. The complexity of the lung tumor microenvironment (TME) is the main factor leading to misdiagnosis. Chronic respiratory diseases, such as asthma, emphysema, and bronchitis, are still diagnosed based on respiratory symptoms, medical imaging, and lung function parameters, but they are highly heterogeneous and often overlap [4]. This vague description of underlying disease mechanisms leads to non-specific treatment schemes that may ultimately decrease the effectiveness of treatment for these diseases. In addition, tuberculosis and pneumonia place a substantial economic burden on the patients’ families and society. Therefore, revealing the pathogenesis of respiratory diseases, searching for specific biomarkers, and introducing new therapeutic targets are important strategies to improve the current diagnosis and treatment.

Transcriptomics is a significant advance in combining high-throughput sequencing and bioinformatics to explore biological mechanisms [5]. However, sequencing analysis of bulk tissue obscured individual cell phenotypic and functional differences and could not identify the molecular features of single-cell resolution [6]. With the development of single-cell RNA sequencing (scRNA-seq), an increasing number of cell types and subtypes are being detected and clarified that allow defining the multiple cell types and associated molecular characteristics of the lung, providing a valuable tool for studying the respiratory system [7, 8]. Nevertheless, due to the loss of spatial information caused by cell dissociation in single-cell sequencing, the interactions and functional changes of adjacent cells cannot be described in lung anatomy [9, 10]. Moreover, the relationship between cell state and different cell positions should be clearly elucidated [11].

Since it was proposed in 2016, spatial transcriptomics (ST) has provided a new perspective to decipher physiological and pathological bases [12]. While maintaining the original spatial context, quantitative transcriptome analysis allows the resulting gene expression signatures to correlate with cell spatial localization, physiology, and histology. In addition, ST studies can reveal subcellular RNA distribution patterns to help understanding the biological processes of spatial labelling and regulation. With the development of techniques to collect and process samples for ST, the simultaneous reduction in reagent and sequencing costs, and the increasing potency of computing platforms, the potential to tackle fundamental biological inquiries is steadily expanding. Some related applications of ST have been reviewed [13–15]. However, compared to brain neuroscience, embryonic development, and heart and other organ tissues, ST has rarely been reviewed in respiratory and lung disease research.

This review focuses on the latest iterative technology of ST in recent years and summarizes how these techniques can be applied to the physiological and pathological processes of the respiratory system, such as lung development, lung atlas, lung cancer, and lung injury. Finally, the current challenges and potential development directions are proposed, including high-throughput full-length transcriptome, multi-omics and spatiotemporal omics integration, bioinformatics analysis, etc. By presenting a unique combination of comprehensive disease coverage, in-depth exploration of disease mechanisms, emphasis on spatial heterogeneity, and future directions, this review can provide a distinct and valuable contribution to the field of ST application in respiratory diseases.

## Developments and limitations of ST

Spatial transcriptome methods have developed and emerged rapidly and can be categorized into imaging-based and sequencing-based methods according to detection strategies. Previous reviews have elaborated on the principles and classification of ST [10, 16, 17]. This article focuses on the innovative forms and variants of these technologies.

### Imaging-based ST strategies

**Fig. 1:**
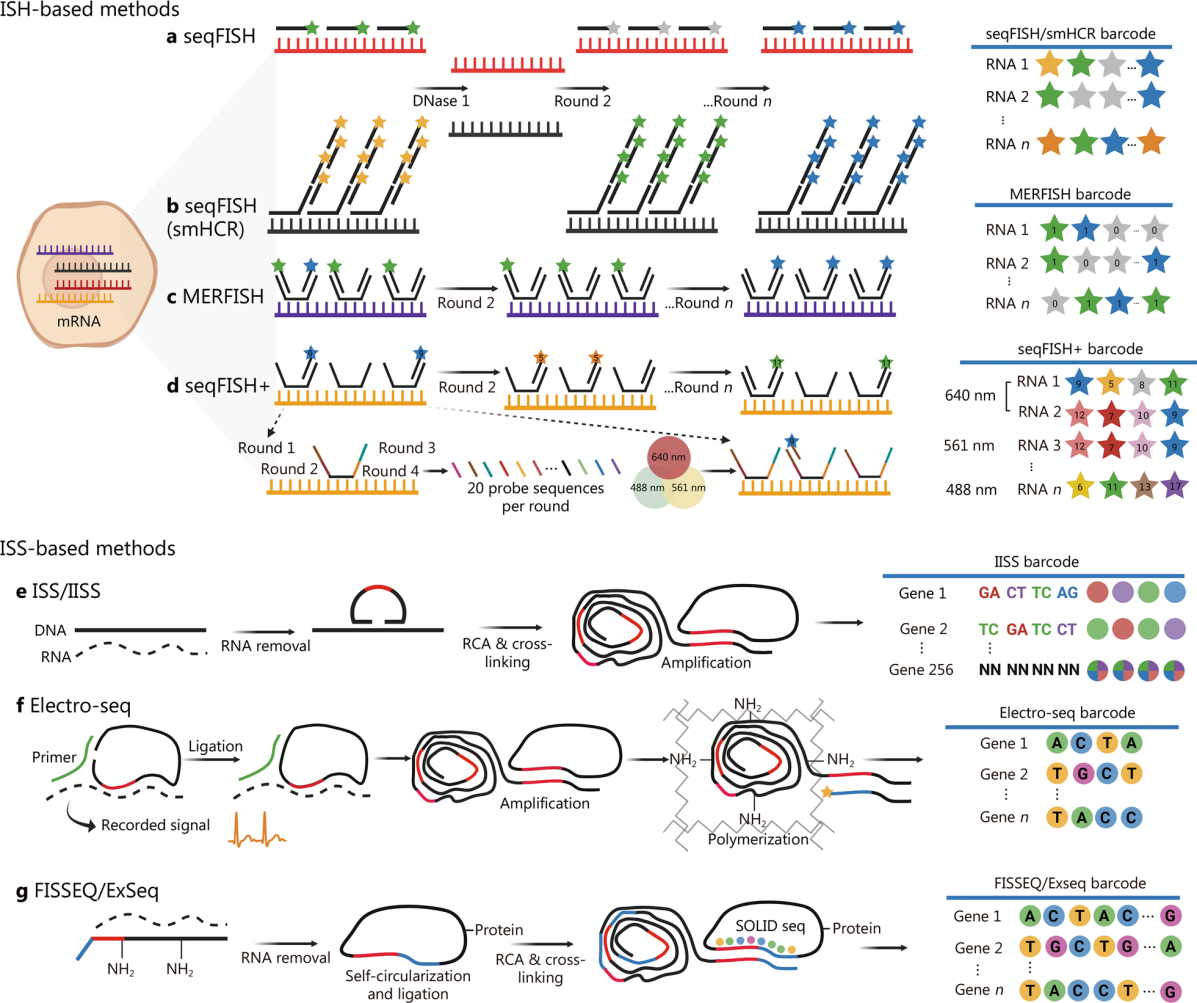
Imaging-based ST strategies. **a** seqFISH decodes transcripts in space by sequential staining/imaging cycles. **b** Compared to seqFISH, smHCR can achieve ~ 20 fold signal amplification to detect single mRNA in situ. **c** MERFISH implements thousands of RNA imaging using a combinatorial FISH labeling with encoding schemes that correct errors. **d** seqFISH+ performs in situ RNA imaging using 20 probes in four-wheel coding. **e** ISS-methods by sequencing by ligation (ISS, IISS). **f** Electro-seq combines bioelectronics with ISS enabling electrophysiological and gene expression profiling. **g** Non-targeted sequencing, such as FISSEQ and ExSeq, enables unbiased covering sequencing of the whole transcriptome. ST spatial transcriptomic, FISH fluorescence in situ hybridization, seqFISH+ sequential FISH+, smHCR single-molecule hybridization chain reaction, MERFISH multiplexed error-robust FISH, ISH in situ hybridization, ISS in situ sequencing, IISS improved in situ sequencing, FISSEQ fluorescent in situ sequencing, Electro-seq in situ electro-sequencing

Image-based ST technologies include fluorescence in situ hybridization (ISH)- and in situ sequencing (ISS)-based methods. The advent of high-resolution microscopy and single-molecule fluorescence in situ hybridization (smFISH) has made it possible to quantify the subcellular resolution of transcripts in situ [18]. A unique fluorescently labelled probe binds to RNA, allowing the localization of individual molecules. The variant RNA scope of this technology is commercially available [19], and strategies such as multi-round hybridization, imaging, and probe dissection have widely been applied, such as sequential FISH (seqFISH) [20, 21] (Fig. 1a). Hybridization chain reactions (HCR) based on isothermal amplification have also been applied to solve the problem of high autofluorescence, namely smHCR [22] (Fig. 1b). To overcome probe hybridization errors and read errors, a barcode allocation scheme, multiplexed error-robust FISH (MERFISH), has been developed and widely used in single-cell transcription localization and ST at the tissue level (Fig. 1c). On this basis, sequential fluorescence in situ hybridization (seq-FISH +) [23], ouroboros smFISH (osmFISH) [24], and signal amplification by exchange reaction (SABER) [25] are proposed to solve the molecular crowding in the imaging process. Among them, seq-FISH + used 20 probes and 3 excitation lights to analyse 10,000 genes (Fig. 1d). Enhanced electric FISH (EEL FISH) combines electrophoresis-aided large tissue RNA sampling and multiplexed FISH for transcription imaging of thick tissue, reducing data collection time [26]. Recently, expansion FISH (exFISH) [27] and expansion-assisted iterative fluorescence in situ hybridization (EASI-FISH) [28] were used for the three-dimensional (3D) resolution of gene expression in tissues using hydrogel expansion. Overall, due to the expensive and time-consuming nature of hybridization techniques and additional challenges, such as background fluorescence in tissues, ISH-based methods have thus far been limited to research on cell and tissue culture.

ISS-based methods are categorized into targeted and untargeted mRNA detection. One of the earliest targeted in situ sequencing techniques, ISS (Fig. 1e), generated signals through padlock probes and rolling circle amplification (RCA), enabling the expression of 256 RNA transcripts in single-round hybridization and was commercialized as Cartana [29]. Then BOLORAMIS (barcoded oligonucleotides ligated on RNA amplified for multiplexed and parallel in situ analyses) solved the problem of low detection efficiency of ISS by introducing the DNA ligase SplintR ligase [19]. Hybridization-based ISS (HybISS) allows a barcoding system to improve in situ detection and removes the limitations of the sequence-by-ligation chemistry of ISS. BaristaSeq utilizes synthetic chemical sequencing technology to display higher signal-to-noise ratios through multiple rounds of imaging and perform specific detection of RCA products [30]. In a preprint study in 2023, Tang et al. [31] proposed improved ISS (IISS), which developed an improved combinatorial probe anchor ligation chemistry using a 2-base encoding strategy for barcode interrogation, improving the signal strength and specificity of ISS (Fig. 1e). Moreover, a recent study integrated electrochemistry and ISS (Electro-seq) to correlate cell electrophysiology with gene expression at the single-cell level and identify changes in the gene expression profile during myocardial cell development [32] (Fig. 1f).

On the other hand, spatially-resolved transcript amplicon readout mapping (STARmap) bypasses cDNA synthesis and uses SNAIL probes and sequencing with error-reduction by dynamic annealing and ligation (SEDAL) to identify gene identifiers. By combining hydrogel histochemistry, it analyses tissue samples in 3D tissues rather than a single 2D pattern [33]. This year, the team further updated STARmap, or STARmap PLUS, and realized the joint detection of transcriptome and protein in a mouse model of Alzheimer’s disease [34].

Fluorescent in situ sequencing (FISSEQ) is a representative method in non-targeted sequencing that can achieve unbiased coverage of the whole transcriptome. However, its random primers lead to low detection efficiency and involve complex enzymatic reactions [35] (Fig. 1g). Relevant reagents and instruments have been produced and commercialized [36]. Based on FISSEQ, untargeted expansion sequencing (ExSeq) combines expansion microscopy with ISS, using an amplified hydrogel to anchor RNA and generate optical barcodes, which have been used for gene analysis in Drosophila embryos and mouse brains [37] (Fig. 1f).

### Sequencing-based ST strategies

**Fig. 2:**
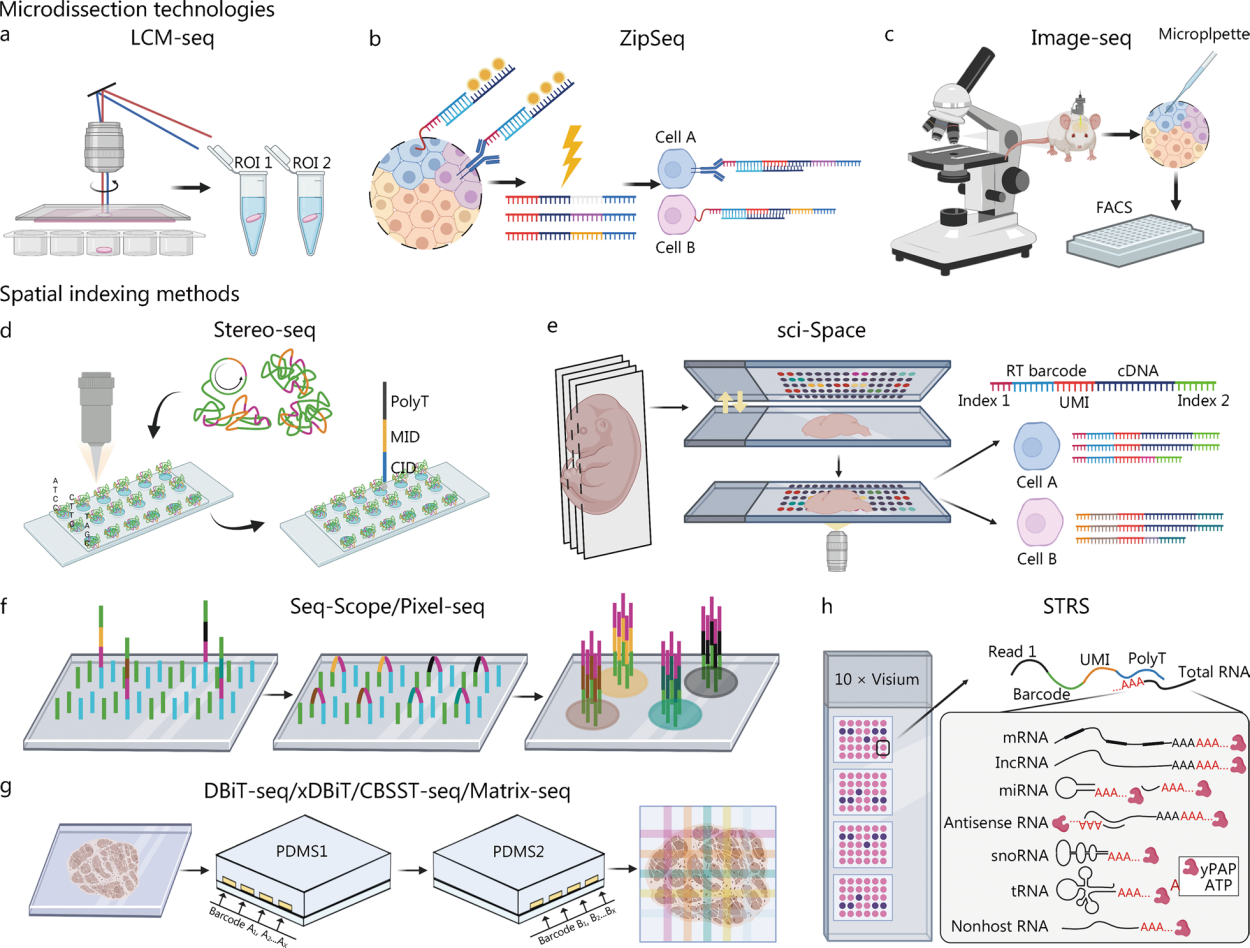
Sequencing-based ST strategies. **a** LCM-seq integrates LCM and scRNA-seq to realize regional sequencing. **b** ZipSeq uses light activation tags for labeling, isolation, and scRNA-seq of ROIs. **c** Image-seq allows location-specific live cells to be harvested for sequencing by living microscope. **d** Stereo-seq utilizes DNA nanoballs with spatial barcodes for transcription spatial localization. **e** sci-Space uses spatial barcodes for imaging, labeling and transcriptome sequencing of nuclei in tissue slices. **f** Seq-Scope and Pixel-seq based on illumina clustering and sequence reading. **g** DBiT-seq utilizes microfluidic channels for orthogonal coding, and similar techniques include xDBiT, CBSST-seq and Matrix-seq. **h** STRS utilizes the 10 × Visium platform for total transcriptome analysis. ST spatial transcriptomic, LCM laser capture microdissection, scRNA-seq single cell RNA sequencing, ROI regions of interest, Stereo-seq spatio-temporal enhanced resolution omics-sequencing, Pixel-seq polony-indexed library-sequencing, DBiT-seq deterministic barcoding in tissue for spatial omics sequencing, CBSST-seq cross-amplified barcodes on slides for spatial transcriptomics sequencing, STRS spatial total RNA-sequencing, FACS fluorescence activated cell sorting, UMI unique molecular identifier, MID molecular identifiers, CID coordinate identity, mRNA messenger RNA, lncRNA long noncoding RNA, miRNA microRNA, snoRNA small nucleolar RNA, tRNA transfer RNA, ATP adenosine triphosphate, yPAP yeast poly(A) polymerase, xDBiT multiplexed deterministic barcoding in tissue

Combining the next-generation sequencing (NGS) platform and spatial information significantly improves the throughput of ST and unbiased retrieval of transcripts compared to image-based methods. A method based on laser capture microdissection (LCM) and scRNA-seq was developed to allow a spatially unbiased analysis of the transcriptome and classification and sequencing of regions of interest (ROI) under microscopic guidance (Fig. 2a). In addition, Tomo-seq [38], geographical position sequencing (Geo-seq) [39], and proximID [40] were developed to explain the heterogeneity and spatial differences of a small number of cell transcriptomes. At present, optical markers have replaced the traditional physical anatomy, such as NICHE-seq [41], transcriptome in vivo analysis (TIVA) [42], ZipSeq [43] (Fig. 2b), which use patterned illumination and photocaged oligonucleotides to mark ROIs; GeoMx digital spatial profiling utilizes cleavable oligonucleotide tags to quantify the abundance of RNA or proteins in ROIs [44]. A technique, called Image-seq, allows the harvesting of location-specific live cells for sequencing using a living microscope with high sensitivity and transcription coverage but at the cost of reduced throughput [45] (Fig. 2c).

Given the low throughput and capture rate issues of imaging and microdissection technologies, researchers are gradually considering the in situ spatial indexing methods. ST was developed, mRNA location information and expression levels were mapped using spatial barcode and unique molecular identifier (UMI), which was acquired by 10 × Genomics (100 μm), and its capture efficiency (10 × Visium, 55 μm) was further improved [12]. Slide-seq [46] (10 μm) and high-definition ST [47] (HDST, 2 μm) utilizing random barcode beads have been proposed for higher resolution. Slide-seqV2 (10 μm) optimizes library construction and array indexing and demonstrates high capture efficiency for ST sequencing at near-cellular resolution [48]. However, low sensitivity and bead pre-decoding limit their application. Spatial enhanced resolution omics sequencing (Stereo-seq, 0.22 μm) uses random barcode DNA nanospheres deposited in array mode for nanoscale resolution. It has been applied to construct a spatiotemporal transcription atlas of organogenesis [49, 50] (Fig. 2d). sci-Space [51] (200 μm) and XYZeq (a workflow that encodes spatial metadata into scRNA-seq libraries) [52] (500 μm) analyze cells and nuclear spatial coordinates at large scales. However, they cannot provide actual spatial single-cell profiles because they lose cytoplasmic transcription information (Fig. 2e). Seq-Scope (0.5–0.8 μm) directly uses illumina NGS chips to generate spatial barcode arrays, achieving subcellular resolution of spatial barcodes for visualizing the nuclei [53] (Fig. 2f). Similar illumina chemistry, poly-indexed library-sequencing (Pixel-seq, 1 μm), reduces costs 35-fold through repeatable enzyme replication of barcode-patterned gels and improves resolution 200-fold compared to existing methods [54] (Fig. 2f).

Microfluidic channel-based approaches have also been integrated for spatial localization [55]. Deterministic barcoding in tissue for spatial omics sequencing (DBiT-seq) (10 μm) can spatially encode tissues by cross-coding, allowing transcriptome and protein analysis [56] (Fig. 2g). Inspired by this method, multiplexed deterministic barcoding in tissue (xDBiT) [57], cross-amplified barcodes on slides for spatial transcriptomics sequencing (CBSST-seq) [58] and Matrix-seq (a microfluidics-based barcoding strategy) [59] have also been developed and applied, and extended to spatial multi-omics (SM-omics), such as microfluidic indexing based spatial ATAC and RNA sequencing (MISAR-seq) [60] and spatial co-indexing of transcriptomes and epitopes (spatial-CITE-seq) [61]. In addition, spatial total RNA sequencing (STRS) utilizes the 10 × Visium platform to enable the detection of full-spectrum RNA rather than just polyadenylated RNA transcripts [62] (Fig. 2h). A more general challenge for ST based on spatial indexing methods is how to balance mRNA capture efficiency and lateral diffusion. Moreover, large-scale hybridization reverse transcription may lead to the distortion of gene expression.

## Applications of ST to respiratory research

**Fig. 3:**
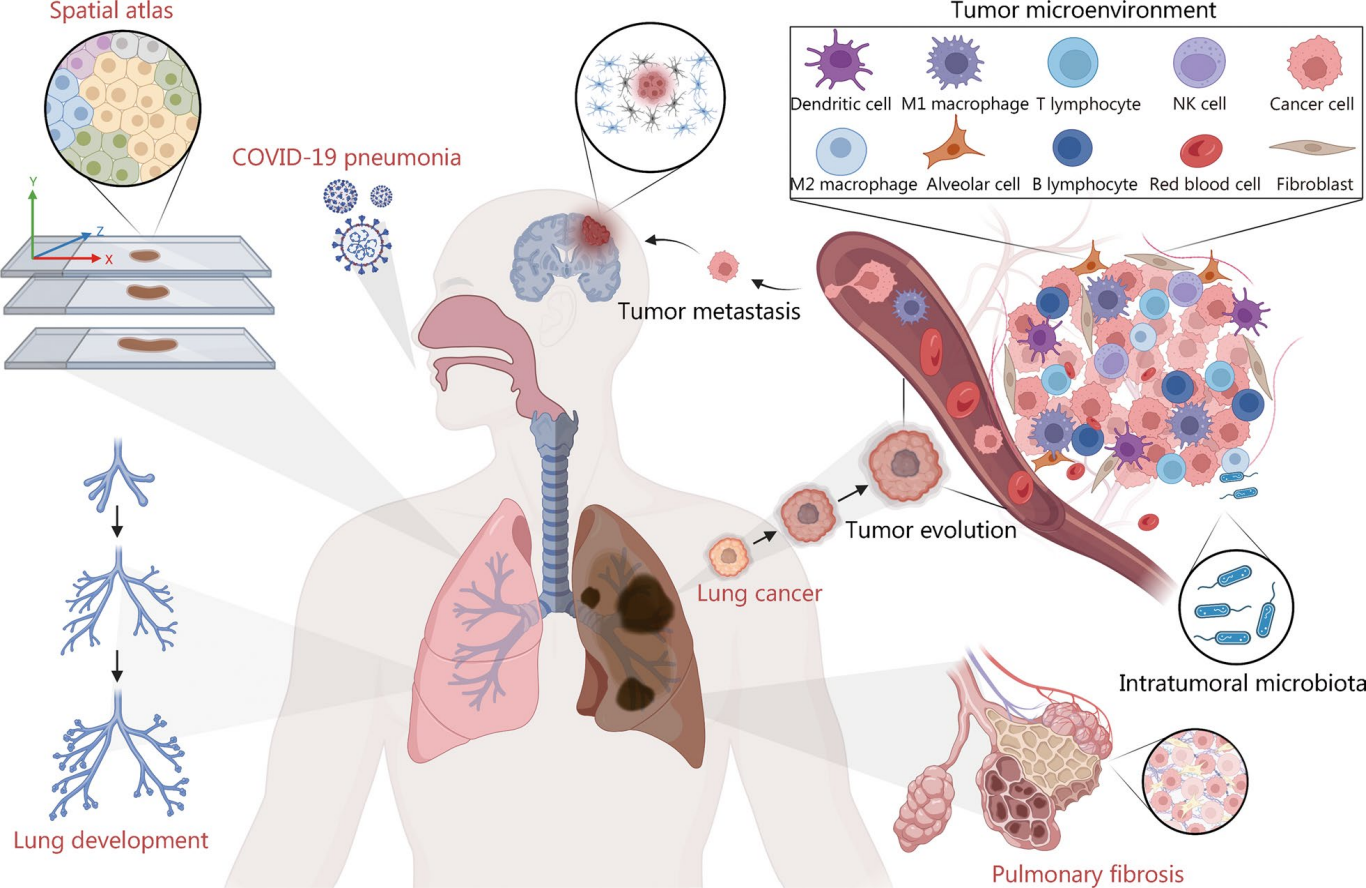
Applications of ST to respiratory research. ST is used to construct lungs spatial atlas and identify cellular composition. In lung cancer, the tumor microenvironment [63] and heterogeneity [64], evolution and metastasis [65], and further localization of intratumoral microbiota origin [66] have been revealed by ST. Other applications of ST include gene expression patterns in lung development [67], and the pathogenesis of pulmonary fibrosis [68] and COVID-19 pneumonia [69]. ST spatial transcriptomic, COVID-19 corona virus disease 2019, NK cell natural killer cell

ST has been widely used in the study of lung development and respiratory disease mechanisms due to the great interest in the molecular structure of the respiratory system. This section summarizes the emerging applications of the respiratory system, such as lung development, lung atlas, lung cancer, and lung injury (Fig. 3) [63–69].

### Lung development

Ljungberg et al. [67] using spatial in situ hybridization attempted to elucidate gene expression patterns in prenatal and postnatal murine lungs to describe the details of lung development at critical stages of alveolarization and improve data for LungMap. A recent study applied in situ hybridization analysis to determine the origin of clonogenic mesenchymal cells in the human lung, especially from an adventitial fibroblast subset. The spatial heterogeneity of mesenchymal cells and their potential characteristics have also been described [70].

### Cellular composition and spatial atlas of lung

The Human Cell Atlas (HCA) aims to establish atlas data of different organs and tissues in healthy individuals at single-cell resolution [71, 72], including the Human Cell Lung Atlas (HCLA), which focuses on the respiratory system. In respiratory research, the cell atlas identifies previously undefined cell catalogues and their phenotypes and interactions, enhancing our understanding of respiratory and lung diseases [73, 74]. Combined with scRNA-seq, many studies have generated lung molecular cell atlases of health and disease, such as LungMAP [75], discovAIR [76], etc. [4, 77, 78]. Researchers have defined 58 different cell types and gene expression profiles in the human lung using droplet- and plate-based scRNA-seq [79]. However, these data are inaccurate due to the lack of spatial context and resolution required to describe the extreme cellular heterogeneity of lungs’ anatomical features. Indeed, a recent study used ST to distinguish 80 cell types and states, including 11 cell populations that had not been annotated in previous lung atlas studies, and define a gland-associated immune niche [80]. Combined with the gene expression profile of scRNA-seq and the spatially resolved transcriptomics on the complete tissue section, Sountoulidis et al. [81] constructed a comprehensive topographic atlas of the early development of human lung, describing the development track leading to significant heterogeneity of lung cell. We believe that the spatial diversity of the lung at the mRNA level is associated with the proteome and, further, with physiological functions. In addition, several in situ hybridization techniques, such as proximity ligation in situ hybridization technology (PLISH) [82] and SCRINSHOT [83] have been developed and used for identifying and localizing lung and airway cell types in mice, including the recently discovered ionocytes. In the distal airways and alveoli, 15 markers were robustly used to identify macrophages and epithelial cells, such as AT1 and AT2 cells, club cells, and neuroendocrine cells.

### Lung cancer

In the past few centuries, tumors have been considered a highly organized “organ” instead of a simple aggregation of abnormally proliferating cells [84]. Lung cancer is the main cause of death from tumor diseases [85]. A significant challenge for medical research is to identify normal cellular trajectory points at the start and progression of lung pathologies and analyse the cellular responses after treatment [86].

#### Lung cancer microenvironment

Immune checkpoint (ICP)-targeted therapy has shown considerable success in lung cancer, including non-small cell lung cancer (NSCLC), lung adenocarcinoma (LUAD), squamous and non-squamous carcinoma. However, studies have shown that the rate of positive responses in patients receiving targeted drugs remains low, with a possibly high degree of immune-related adverse events [87]. In addition, clinical benefits are often prevented by resistance to ICP-targeted drugs in the primary tumor [63]. Increasing evidence suggests that TME is strongly related to tumor development, metastasis, and recurrence and is more critical than ICPs in immune evasion [88, 89]. Therefore, it is urgent to improve the understanding regarding TME to better classify patients and determine new treatment targets to improve prognosis. The cell catalogue transcriptome of the TME in lung tumors is available at single-cell resolution, and its phenotypes and concerted behavior have been described [90]. Spatial transcription analysis can reveal the spatial distribution preference of stromal cells in the lung TME. For example, 10 × Chromium and in situ imaging were used to explore the loss of Tgfbr2 leading to TME remodeling and promoting immune exclusion in a lung cancer mouse model [91]. Tumor-associated macrophages (TAMs), one of the most abundant immune cells in the TME [92], are essential regulators of anti-tumor immunity. However, the mechanisms regulating their abundance in the TME remain to be explored. Larroquette et al. [93] analysed preconditioned tumor samples from advanced NSCLC patients undergoing ICP blocker therapy, reporting that tumor compartment enrichment in TAMs was associated with immunotherapy resistance. A spatial analysis of 78 in situ transcripts from 16 tumor specimens was performed using NanoString GeoMx. The results revealed that the prognostic effect of TAMs in NSCLC was directly related to the distance from tumor cells, and the three significantly up-regulated genes *CD27*, *CCL5*, and *ITGAM* in tumors with high-level TAM infiltration might be potential targets for immunotherapy. Furthermore, applying multiplex immunohistochemistry (mIHC) and digital spatial profiler (DSP) has been observed for TME analysis in samples of NSCLC patients following immune checkpoint inhibitor (ICI) treatment [94].

#### Heterogeneity of lung cancer

Many studies based on scRNA-seq have revealed the heterogeneity of lung cancer cells [95–97]. With advances in spatial transcription analysis and integration of scRNA-seq, researchers have combined specific molecular phenotypes with unknown cell interaction patterns or clinical manifestations to classify tumor cell subgroups. For example, Sinjab et al. [64] identified cell lineages, states, and transcriptomic features that evolved geospatially from normal tissue regions to LUAD, where significant expression of CD24 in epithelial cells drives primary tumor features. Their data provided a spatial atlas of LUAD to identify potential targets for early interception. Zhang et al. [98] used the Visium platform to identify spatial location-specific subclones in the lung squamous cell carcinoma (LUSC). The results showed that the immune cell composition of the tumor subclones was significantly different from the tumor proportion, and the tumor purity was contrary to the trend of tumor epithelial-mesenchymal transition (EMT). The effect of high intratumoral heterogeneity (ITH) on therapy efficacy in advanced lung cancer has recently been reported [99]. The innovative use of DSP has provided compelling evidence for improved prediction of therapeutic outcomes in dual-specificity antibody therapy by integrating genetic information from the stromal region.

#### Development and metastasis of lung cancer

Cancer development involves tumor cells’ adaptation to the environment and is the inevitable and continuous result of life [14]. To date, tracking cancer evolution in humans has focused on DNA mutations. However, genotypes are not necessarily phenotypes [100], and cancer cell populations within a tumor often exhibit significant differences and transcriptional diversity [101, 102]. In this line, lung cancers exhibit more complex molecular and morphological heterogeneity and different combinations of subclone mutations, reducing the reproducibility of lung cancer research and posing a challenge for effective treatment [103]. Zhu et al. [65] integrated RNA-seq and ST techniques and constructed a single-cell spatiotemporal multi-omics atlas of LUAD to explore the dynamic evolution trajectory of early LUAD. The results suggested that LUAD might originate from Clara and AT2 cells, eventually evolving into the UBE2C^+^ cancer cell subpopulation, in which UBE2C mediates the proliferation and metastasis of tumor cells. As LUAD progresses from adenocarcinoma in situ (AIS) to invasive adenocarcinoma (IAC), the spatial distribution of cancer cells may be more important than their type. This finding compensates for the absence of spatial information for immunotyping LUAD by scRNA-seq. Furthermore, studies have reported the mechanisms of brain metastasis (BrMs) in NSCLC based on DSP. These findings highlight the highly immunosuppressive microenvironment associated with BrMs lesions, as compared to primary tumors, characterized by the reduced abundance of B and T cells and increased infiltration of neutrophils, providing a framework for the spatial heterogeneity of BMS in lung cancer and identifying the characteristic genes of metastasis to predict patients’ prognosis [104].

#### Intratumoral microbiota

Lung cancers have a unique microbial composition [105]. Several studies have shown that bacterial populations within tumors are tumor type-specific and might directly regulate cancer initiation and progression [106]. Wong-Rolle et al. [66] obtained spatial macro transcriptome information from 12 lung cancer patients to understand the spatial distribution of the microbiota in lung cancers and its effect on host cell heterogeneity, reporting that bacteria were significantly concentrated in tumor cells, and the content increased from normal tissues and tertiary lymphoid structures (TLS) to tumor cells and peaked in the airway, suggesting that the bacteria in lung cancer may be derived from the airway rather than the intestinal flora. Further gene expression correlation analysis showed that bacterial content positively correlated with the *CTNNB*, *HIF1A*, and *VEGFA* genes. In addition, several signaling pathways related to tumorigenesis also positively correlated with bacterial content in lung cancer. We believe that spatial transcriptome and local interaction group analysis can predict individual tumor behavior and provide useful resources for understanding and reversing lung cancer progression.

### Pulmonary fibrosis

Pulmonary fibrosis is a highly heterogeneous end-stage pathological change of the lung, whose pathogenesis has not been fully elucidated [107, 108]. The first published study of pulmonary fibrosis using ST focused on epithelial cells [68], demonstrating the unique molecular signature of epithelial cell/fibroblast foci sandwiches from typical interstitial pneumonia/idiopathic pulmonary fibrosis (IPF) patients. The pathogenesis of IPF is associated with epithelial dysfunction. Another study used the GeoMx DSP platform to explore the transcriptional differences between fibroblastic foci and fibrous and normal areas in IPF cases and identified new fibrogenic biomarkers expressed in fibroblastic foci [109]. The spatiotemporal analysis brings hope for the development of pulmonary fibrosis. For example, Shi et al. [110] explored the spatiotemporal distribution of heterogeneous fibroblasts in the progression of secondary pulmonary fibrosis due to silica inhalation. The results showed that GREM1, as a driving factor causing inflammation, is involved in changes in this pulmonary condition and may be a potential target for the early treatment of silicosis.

### Pneumonia

The corona virus disease 2019 (COVID-19) pandemic, induced by severe acute respiratory syndrome coronavirus 2 (SARS-CoV2), has caused millions of cases of severe acute respiratory illness worldwide [111, 112], the pathogenesis of which is not fully understood, and the host response to its infection should be better defined. ST provides new insights into identifying cell types and elucidating heterogeneity after SARS-CoV2 infection, as shown in Table 1 [69, 113–124]. The Broad Institute, in collaboration with Harvard Medical School and others, created the post-infection lung spatial transcriptome atlas [69], revealing extensive remodelling of the lung epithelial, immune, and stromal compartments and mapping cell types and genes associated with disease severity. Analysis of COVID-19-infected patients highlighted two stages of the disease that lead to death in patients. Desai et al. [113] showed that RNA levels were associated with disease duration, reporting significant spatiotemporal heterogeneity in viral load and immune response. It is estimated that COVID-19 infection will lead to various complications and post-acute sequelae of SARS-CoV2 (PASC). Dinnon et al. [114] constructed a mouse model and identified the transcriptional profiles of acute and chronic disease stages using RNA-ISH and GeoMx DSP, providing strategies for testing and improving the “long COVID”. Moreover, DSP data obtained from lung tissues in areas affected by acute respiratory distress syndrome (ARDS) induced by SARS-CoV2 and H1N1 indicate unique transcriptional signatures, thus identifying novel therapeutic targets [115].

**Table 1:** Study of mechanisms and complications of COVID-19 using spatial transcriptomics

Strategy	Species	Application	Ref.
RNA-ISH and GeoMx DSP	Human	Spatiotemporal heterogeneity of SARS-CoV-2 infection	[113]
RNAscope and GeoMx DSP	Human	Revealed distinct ACE2 expression loci	[116]
GeoMx DSP	Human	Mechanism of ARDS by SARS-CoV-2	[115]
RNAscope and GeoMx DSP	Human	Generate COVID-19 related lung spatial atlas	[69]
GeoMx DSP	Human	Identification of COVID-19 therapeutic target	[117]
RNAscope and GeoMx DSP	Human	Investigation of the pathogenesis of SARS-COV-2	[118]
GeoMx DSP	Human	SARS-COV-2 infection up-regulates inflammatory response in COPD patients	[119]
RNA-ISH and GeoMx DSP	Mouse	Mechanism of PASC	[114]
RNAscope	Human	Revealed the responses to SARS-CoV-2-induced exudative DAD	[120]
Visium	Mouse	A dynamic change in the location of T helper cells as well as their corresponding chemokines	[121]
RNAscope and GeoMx DSP	Human	Construction of immune-mediated histopathology model of COVID-19	[122]
Visium	Human	The interaction between chemokines and receptors in the pathogenesis of COVID-19 lung niche	[123]
Visium, RNAscope and GeoMx DSP	Human	Map Immune Responses to SARS-CoV-2	[124]

*RNA-ISH* RNA-in situ hybridization, *GeoMx DSP* GeoMx digital spatial profiler, *ACE2* angiotensin converting enzyme 2, *COVID-19* corona virus disease 2019, *SARS-CoV2* severe acute respiratory syndrome coronavirus 2, *ARDS* acute respiratory distress syndrome, *COPD* chronic obstructive pulmonary diseases, *PASC* post-acute sequelae of SARS-CoV2, *DAD* diffuse alveolar damage

### Other respiratory diseases

In situ sequencing and capture techniques have been applied to the mechanistic study and therapeutic target discovery of tuberculosis [125–127] and influenza infections [128]. For example, a study used mice infected with H1N1 as the model for studying ARDS [129], demonstrating that over-activated fibroblasts could produce extracellular matrix remodelling enzymes, thereby promoting the infiltration of immune cells and leading to compromised lung function. Many studies have evaluated other respiratory diseases, such as pulmonary arterial hypertension [130], asthma [131], and chronic obstructive pulmonary disease (COPD) [132], using scRNA-seq. The introduction of space omics technology is expected to deepen our understanding of respiratory diseases.

## Challenges and perspectives

**Fig. 4:**
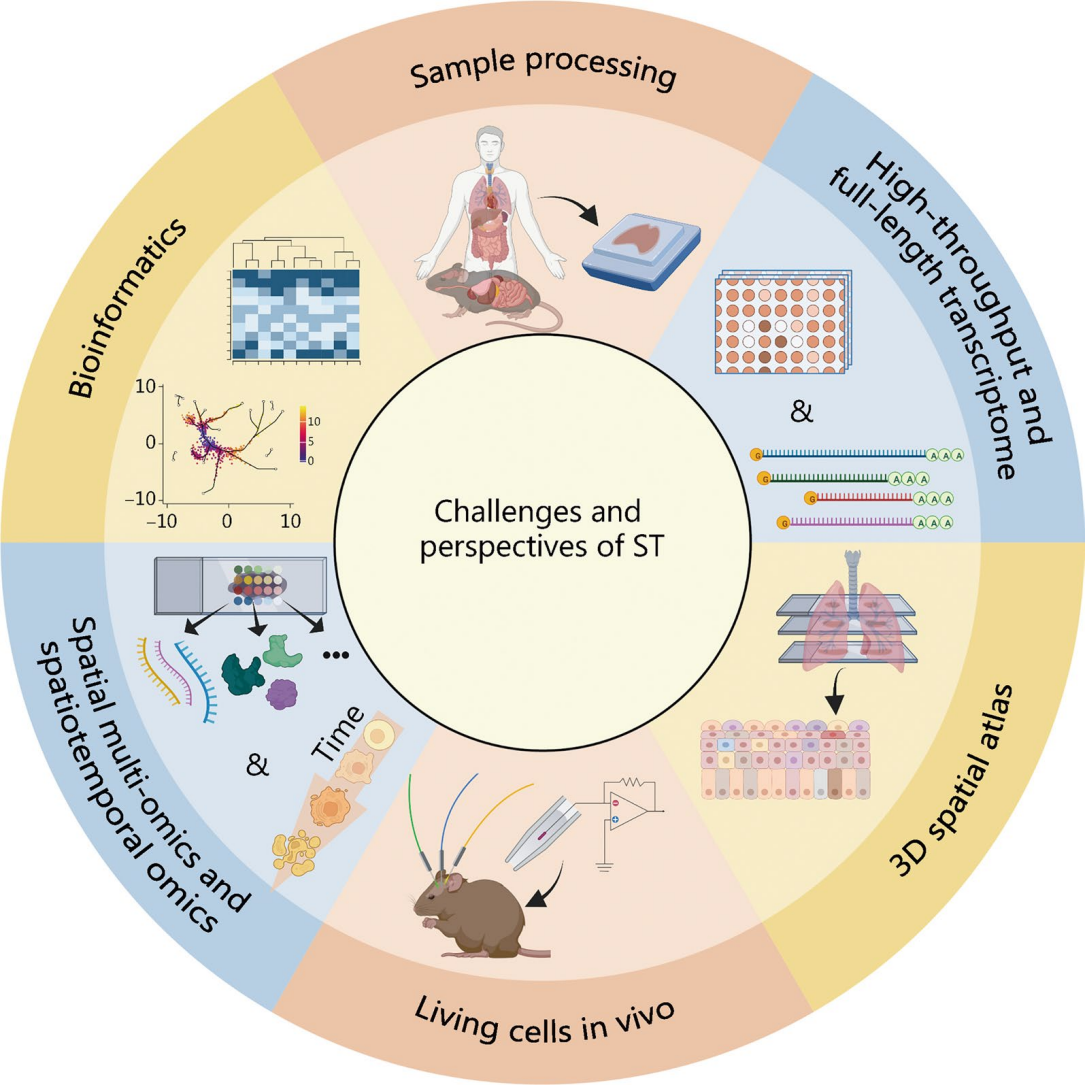
The challenges and perspectives of spatial transcriptomics (ST). ST moves toward high-throughput and full-length transcriptome, in vivo analysis of living cells, and integrates multi-omics and spatiotemporal omics, enabling the construction of 3D spatial atlas with the help of sample processing and bioinformatics tools and providing powerful new techniques for interrogating tissue structure and function

Although ST and related frontier bioinformatics analysis have entirely changed the research on complex organs and tissues and brought great hope for developing systems and disease mechanisms, several challenges should still be addressed to develop its potential beyond these (Fig. 4).

### Preparation and treatment of samples

The stability and availability of pre-sequencing samples might be a major obstacle limiting ST. Any intact tissue containing mRNA is suitable for the spatial transcriptome [10]; however, the sample preparation regimen should be further optimized according to the characteristics of different tissues. Morphological differences between tissue types should also be considered. For example, single cell/tissue spots significantly affect transcription levels, and hyperpigmentation in skin cell samples can negatively affect image acquisition due to light absorption [133]. Similarly, lung tissues with alveolar sacs must be handled carefully when frozen [134]. In addition, the sample collection, storage, and processing methods have measurement deviations from the original transcriptional data. One study explored the temporal and spatial gene expression map of the developing human heart [135]. Heart tissue was chopped up and cultured in a suspension using trypsin and collagenase to produce individual cells. Some studies have applied exogenous reagents for isolation [136, 137]; however, they have hardly mentioned whether physicochemical-based isolation and unnatural processing would affect the cells’ transcription levels, reducing the reliability of the generated scRNA-seq data [138]. Concerning spatial transcriptome technologies, in situ capture and sequencing-based strategies [51, 139, 140] have achieved tissue dissection-free sequencing, significantly reducing the risk of sample preparation. However, the effect of tissue slice preparation on the transcriptome should also be considered from a macroscopic perspective. Therefore, further innovations are necessary in sample preparation. Ideally, the upstream processing of tissue sections for sequencing is automated and accessible to nonprofessional users.

A growing body of evidence suggests that ST can potentially aid in clinical diagnosis, understanding human disease, and making proper decisions concerning medicines [141]. Formalin-fixed paraffin-embedded (FFPE) tissue blocks are the gold standard of human tissue preservation methods for clinical diagnosis. The rapid development of space transcriptomics is compatible with FFPE blocks, a breakthrough in the in-depth pathological analysis of more than one billion FFPE sections in the sample library [142, 143]. Unfortunately, RNA molecules in samples are often seriously degraded; therefore, a platform that can analyze FFPE samples is urgently needed. The 10 × Genomics Visium and NanoString DSP have demonstrated their compatibility with FFPE blocks [144, 145]. The Visium Cytassist, launched in late 2022 by 10 × Genomics, enables automated transfer analysis of tissue sections in 30 min, with simultaneous transcriptome and protein analysis of one FFPE sample [146]. Nevertheless, since FFPE tissue blocks are usually stored in a fixing solution, and the time from biopsy to fixation may differ between samples, the integrity of RNA will also be much lower than that of fresh frozen tissue. In addition, since the quality of data may depend on the specific sample, its reliability and robustness should be improved before clinical applications.

### High-throughput full-length transcriptome

The ST has solved the problem of losing spatial location information by cell dissociation in the scRNA-seq method. However, apart from spatial addressing, the transcription coverage and depth of these methods are still in their early development stages. Most ST methods can only retrieve single-ended transcripts rather than full-length ones [147]. The residual sequence in the polyadenylated RNA molecule and the spectrum of non-polyadenylated transcripts have not been detected, hindering the study of the immune cell receptor spectrum and alternative splicing (AS). Microdissection-based methods exhibit ideal coverage but suboptimal throughput and spatial resolution of transcription, with a trade-off between the coverage of tissue sections and the detection sensitivity of transcripts. ISH-based methods exhibit excellent spatial resolution and detection efficiency; however, they are difficult to use for large tissue sections and high throughput. ISS has a high resolution but sacrifices the transcript capture depth. Recently, a method of vast transcriptome analysis of single cells by dA-tailing was proposed [148], namely, VASA-seq, which is the only single-cell sequencing technology to combine high sensitivity, full-length transcriptome coverage, and high throughput. However, VASA-seq cannot obtain the spatial location information of cells. In addition, although combining high-throughput microfluidics and barcode index improves the throughput, the quality of RNA might decrease due to cell fixation and permeability treatment. In addition, the cell/bead co-encapsulation efficiency should be improved. Therefore, techniques with higher gene coverage, lower detection deviation, and higher spatial resolution down to the single-cell level are highly anticipated [55].

### 3D spatial atlas

Currently, most advanced space technologies are limited to displaying cell organization and gene expression on a 2D pattern rather than an actual 3D spatial atlas, which cannot summarize the highly complex spatial cell environment. Tomo-seq performs RNA-seq on different tissue slices to obtain spatial information [149]. Similarly, Geo-seq is combined with the LCM technique to study the transcriptome of small samples with geographical positions. Peng et al. [150] used it to construct a 3D transcriptome atlas of murine embryos and accurately map single ectodermal cells back to their in vivo locations. However, the Geo-seq operation is complicated and requires continuous tissue sections, partitioning by LCM technology, and the sequencing process of database construction [39]. Recently, NanoString’s GeoMx DSP and STRP-seq have been developed to combine bioinformatics tools to reconstruct 3D structural maps such as murine brains [151], and human hearts [152, 153]. However, these technologies are limited by spatial resolution and large sample sizes. We believe that high-precision reconstruction and characterization of 3D structures will further provide a solid foundation for clinical application and research.

### Living cells/tissue in vivo

The current ST methods cannot be applied to in vivo histological research on living cells because they investigate only cell snapshots at a certain time in fixed samples. The observed gene expression profile may only be the product of expression heterogeneity [154]. These technologies limit the cell to the active transcriptional state at a single time point, while other cells with similar functions may be dormant. Therefore, their ability to correctly infer cells at the individual gene level and time scale is still controversial. Some research groups have tried to study the transcriptome of living cells in vitro. For example, TIVA has pioneered the capture of mRNA in living cells for transcriptional analysis [42]. However, this method is currently not applicable to the analysis of many cells. ZipSeq marks DNA codes (Zipcodes) on living cells of intact tissue using photocaged oligonucleotides and specific patterned illumination to explore its spatial heterogeneity [43]. However, its low spatial resolution limits its application scope. Similarly, Live-seq is the first to realize continuous observation of whole gene expression in living cells [155]. However, some problems remain, such as inapplicability in vivo and the limitation of multiple sampling. Therefore, the next research direction is how to infer the future or correlate past events with current gene expression, realize the exploration of spatial omics in living cells or tissues in vivo, and track mRNA dynamics in real time with minimal cell disturbance.

### SM-omics and spatiotemporal omics

ST is progressing rapidly towards multi-omics data and achieving single-cell resolution. This advancement enables the retrieval of comprehensive information encompassing splicing variation, genetic and epigenetic changes, proteomics, and time-dimension data, all within a single experimental setup [15]. This will assist in understanding cell–cell interactions and the overall cell phenotype/state to solve the tissue function from multiple spatial scales [156]. As mentioned previously, the most robust strategy to obtain a more comprehensive multi-omics profile is to process continuous tissue sections, in which each section is queried by different omics. However, continuous tissue section sampling might give rise to sample heterogeneity deviation.

Researchers have significantly advanced in developing of various single-cell multi-omics technologies, as exemplified in Table 2 [157–181]. Furthermore, multiple databases have been specifically curated to cater to human single-cell omics, providing comprehensive analytical tools for data analysis and interpretation [182, 183]. On this basis, SM-omics technologies, including multi omic single-scan assay with integrated combinatorial analysis (MOSAICA) [184], SM-omics [185], spatial molecular imaging (SMI) [186], DBiT-seq [56] and spatial protein and transcriptome sequencing (SPOTS) [187], which retain spatial information coordinates, have been developed. Developing new technologies such as mIHC [188] and cytometry by time of flight (CyTOF) [189], also promotes joint analysis with ST. At present, SM-omics is on Nature’s annual technologies list in 2022. Nevertheless, SM-omics sequencing is still in its infancy. Splicing variants have traditionally been difficult to detect at the RNA level because most techniques are based on single-ended transcription analysis. At the protein level, the analysis of targeted proteins is limited by the number of available variants, such as fluorescent dyes, barcodes, or antibodies, and is limited to the analysis of targeted proteins. In addition, histone modification, chromatin accessibility, and metabolomics research still lack spatial counterparts. The integrated approach requires high-quality acquisition of multiple parameters, such as gene coverage, throughput, accuracy, and sequencing depth. Finally, how to achieve actual single-cell resolution should also be considered.

**Table 2:** Single cell multi-omics analysis

Method	Year	Application	Ref.
Transcriptome and genome			
G&T-seq	2015	Detection of SNVs and cell chromosome rearrangement	[157]
DR-Seq	2015	DNA copy-number variations within the cancer genome	[158]
SIDR	2018	Copy-number and single-nucleotide variations	[159]
TARGET-seq	2019	The distinct transcriptional signatures of tumor genetic subclones	[160]
DMF-DR-seq	2022	The genome variation-induced abnormal transcriptome expression	[161]
Transcriptome and epigenome			
scM&T-seq	2016	Associations between transcriptional and epigenetic variation	[162]
scMT-seq	2016	Correlation between DNA methylation and gene transcription	[163]
scTrio-seq	2016	Epigenetic, and transcriptomic heterogeneity in hepatocellular carcinomas	[164]
sc-GEM	2016	Epigenetic variations within and between different cell types	[165]
scNMT-seq	2018	Epigenome interactions during a developmental trajectory	[166]
snDrop-seq and scTHS-seq	2018	Identification of region-specific neuronal and non-neuronal cell types	[167]
sciCAR	2018	Compare the pseudotemporal dynamics of chromatin accessibility	[168]
Paired-seq	2019	Analyze the dynamic and cell-type-specific gene regulatory programs	[169]
SNARE-seq	2019	Reconstruct the transcriptome and epigenetic landscapes of cells	[170]
scCAT-seq	2019	Regulatory relationships between cis-regulatory elements and the target genes	[171]
Transcriptome and proteome			
PLAYR	2016	The interplay between transcription and translation	[172]
PEA/STA	2016	Proximity extension assays and complementary DNA synthesis	[173]
CITE-seq	2017	Cellular indexing of transcriptomes and epitopes	[174]
REAP-seq	2017	The costimulatory effects of a CD27 agonist on human CD8^+^ lymphocytes	[175]
Apt-seq	2018	Differentiate distinct cell types	[176]
ECCITE-seq	2019	Clonotype-aware multimodal phenotyping of cancer samples	[177]
INs-seq	2020	Immunosuppressive role of Trem2 in cancer	[178]
SCITO-seq	2021	Cell surface protein abundance	[179]
inCITE-seq	2021	Identification of gene regulatory targets of nuclear proteins in tissues	[180]
Multi-Paired-seq	2022	Dynamic expression and correlations between mRNAs and proteins in individual cells	[181]

*G&T-seq* genome and transcriptome sequencing, *DR-seq* gDNA-mRNA sequencing, *SIDR* simultaneous isolation of genomic DNA and total RNA, *DMF-DR-seq* digital microfluidics gDNA-mRNA sequencing, *scM&T-seq* single-cell genome-wide methylome and transcriptome sequencing, *scMT-seq* single-cell methylome and transcriptome sequencing, *scTrio-seq* single-cell triple omics sequencing, *sc-GEM* single-cell analysis of genotype, expression and methylation, *scNMT-seq* single-cell nucleosome, methylation and transcription sequencing, *snDrop-seq* single-nucleus droplet-based sequencing, *scTHS-seq* single-cell transposome hypersensitive site sequencing, *sciCAR* single-cell combinatorial indexing of chromatin accessibility and mRNA, *Paired-seq* parallel analysis of individual cells for RNA expression and DNA accessibility by sequencing, *SNARE-seq* single-nucleus chromatin accessibility and mRNA expression sequencing, *scCAT-seq* single-cell chromatin accessibility and transcriptome sequencing, *PLAYR* proximity ligation assay for RNA, *PEA/STA* proximity extension assays/specific (RNA) target amplification, *CITE-seq* cellular indexing of transcriptomes and epitopes by sequencing, *REAP-seq* RNA expression and protein sequencing assay, *Apt-seq* aptamers and single cell sequencing, *ECCITE-seq* expanded CRISPR-compatible cellular indexing of transcriptomes and epitopes by sequencing, *INs-seq* intracellular staining and sequencing, *SCITO-seq* single-cell combinatorial indexed cytometry sequencing, *inCITE-seq* intranuclear cellular indexing of transcriptomes and epitopes, *SNVs* single nucleotide variants, *DNA* deoxyribonucleic acid, *mRNA* messenger RNA

On the other hand, gene expression in biological systems is highly dynamic [190], and analysing cellular interactions and regulatory mechanisms from temporal and spatial dimensions assists in understanding the rules in complex systems, namely, spatiotemporal omics. RNA metabolic labelling strategies were introduced in RNA-seq to distinguish new mRNA from old ones, such as 4-thio-uridine (4sU) [191] and 5-ethynyl-uridine (5-EU) [192]. In addition, RNA timestamps bind RNA-seq to infer the “age” of RNA in hours [193]. However, these methods are only suitable for describing short time scales or points in time characteristics of cells. Therefore, continuous analysis and spatial localization of the same cell make spatiotemporal omics possible, such as “high-throughput Patch-seq” [194] and “Live-seq with spatial context” [155].

### Bioinformatics support

Increased spatial dimensionality, data volume, and complexity present formidable challenges during the data analysis phase. Numerous bioinformatics tools employed for scRNA-seq analysis can be readily adapted for spatial analysis, encompassing deconvolution, clustering, cell type annotation [195, 196], and other essential processing steps [197]. However, it ignores spatial location and structural characteristics; therefore, the current bioinformatics pipeline must be improved to analyze the unique properties of ST. Bioinformatics analysis software for ST is emerging endlessly. Trendseek [198], SpatialDE [199], graph laplacian-based integrative single-cell spatial analysis (GLISS), and SpaGCN [200] have been developed to analyze the relationships between spatial location and gene expression. Methods for studying the interactions between cells include Graph Convolutional Neural networks for Genes (GCNG), spatial variance component analysis (SVCA) [201], novaSpaRc [202], and SpaOTsc [203]. Methods like stLearn, BayesSpace [204], spatial clustering using the hidden Markov random field based on empirical bayes (SC-MEB) [205] and MULTILAYER [206] are used for spatial clustering. It is anticipated that advancements in spatial omics technologies will result in the proliferation of analytical computing tools, facilitating the harmonization of data across diverse platforms and fostering the integration of information tools such as machine learning and image segmentation. This integration is crucial for a deeper understanding of intricate spatial structures and expanding availability to a wide array of available data sources [207].

With the exception of commercial platforms like 10 × Visium, Stereo-seq, and GeoMx DSP, the majority of the aforementioned ST techniques are generally confined to laboratory settings. This limitation is expected, given the recent publication of these methods and the high costs associated with translating experiments into diagnostic applications. Standardizing experimental procedures and data analysis pipelines is anticipated to facilitate the commercialization and widespread accessibility of spatial omics analysis techniques. Moreover, ongoing efforts to integrate automated sample processing, 3D structure, deep section scanning, and time series data will further advance this field by revealing new cell structures and expanding our understanding of biological processes. Given that the future progress of ST necessitates the convergence of multiple disciplines, close collaboration is imperative between researchers in bioinformatics analytics, automation devices, clinical translational research, and biomedical fields [208].

## Conclusions

This review presented cutting-edge technologies in ST and their applications to organ/tissue physiological and pathological processes. As a newer iteration of scRNA-seq, the field of ST is expanding rapidly, significantly improving our understanding of developmental biology and pathogenesis and transforming our ability to diagnose, understand, and treat diseases. With this growing panoply and collaborative efforts of bioinformatics, engineering, and SM-omics, we expect to obtain high-throughput molecular information concerning full-length transcriptomes when the cost significantly decreases. These data will provide a more comprehensive view for clarifying interactions between cell biological mechanisms within tissue ecosystems.

## Data Availability

Data availability is not applicable to this article as no new data were created or analyzed in this study.

## Abbreviations

ACE2: Angiotensin converting enzyme 2
AIS: Adenocarcinoma in situ
Apt-seq: Aptamers and single cell sequencing
ARDS: Acute respiratory distress syndrome
AS: Alternative splicing
ATP: Adenosine triphosphate
BOLORAMIS: Barcoded oligonucleotides ligated on RNA amplified for multiplexed and parallel in situ analyses
BrMs: Brain metastasis
CBSST-seq: Cross-amplified barcodes on slides for spatial transcriptomics sequencing
CID: Coordinate identity
CITE-seq: Cellular indexing of transcriptomes and epitopes by sequencing
COPD: Chronic obstructive pulmonary diseases
COVID-19: Corona virus disease 2019
CyTOF: Cytometry by time of flight
DAD: Diffuse alveolar damage
DBiT-seq: Deterministic barcoding in tissue for spatial omics sequencing
DMF-DR-seq: Digital microfluidics gDNA-mRNA sequencing
DNA: Deoxyribonucleic acid
DR-seq: GDNA-mRNA sequencing
DSP: Digital spatial profiler
EASI-FISH: Expansion-assisted iterative fluorescence in situ hybridization
ECCITE-seq: Expanded CRISPR-compatible cellular indexing of transcriptomes and epitopes by sequencing
EEL FISH: Enhanced electric FISH
EMT: Epithelial-mesenchymal transition
exFISH: Expansion FISH
ExSeq: Expansion sequencing
FACS: Fluorescence activated cell sorting
FFPE: Formalin-fixed paraffin-embedded
FISSEQ: Fluorescent in situ sequencing
GCNG: Graph Convolutional Neural networks for Genes
Geo-seq: Geographical position sequencing
GLISS: Graph laplacian-based integrative single-cell spatial analysis
G&T-seq: Genome and transcriptome sequencing
HCA: Human Cell Atlas
HCLA: Human Cell Lung Atlas
HCR: Hybridization chain reaction
HDST: High-definition ST
HybISS: Hybridization-based ISS
IAC: Invasive adenocarcinoma
ICI: Immune checkpoint inhibitor
ICP: Immune checkpoint
IISS: Improved ISS
inCITE-seq: Intranuclear cellular indexing of transcriptomes and epitopes
INs-seq: Intracellular staining and sequencing
IPF: Idiopathic pulmonary fibrosis
ISH: In situ hybridization
ISS: In situ sequencing
ITH: Intratumoral heterogeneity
LCM: Laser capture microdissection
lncRNA: Long noncoding RNA
LUAD: Lung adenocarcinoma
LUSC: Lung squamous cell carcinoma
MERFISH: Multiplexed error-robust FISH
MID: Molecular identifiers
mIHC: Multiplex immunohistochemistry
miRNA: MicroRNA
MISAR-seq: Microfluidic indexing based spatial ATAC and RNA sequencing
MOSAICA: Multi omic single-scan assay with integrated combinatorial analysis
mRNA: Messenger RNA
NGS: Next-generation sequencing
NK cell: Natural killer cell
NSCLC: Non-small cell lung cancer
osmFISH: Ouroboros smFISH
Paired-seq: Parallel analysis of individual cells for RNA expression and DNA accessibility by sequencing
PASC: Post-acute sequelae of SARS-CoV2
PEA/STA: Proximity extension assays/specific (RNA) target amplification
Pixel-seq: Poly-indexed library-sequencing
PLAYR: Proximity ligation assay for RNA
PLISH: Proximity ligation in situ hybridization technology
RCA: Rolling circle amplification
REAP-seq: RNA expression and protein sequencing assay
RNA-ISH: RNA-in situ hybridization
ROI: Regions of interest
SABER: Signal amplification by exchange reaction
scCAT-seq: Single-cell chromatin accessibility and transcriptome sequencing
sc-GEM: Single-cell analysis of genotype, expression and methylation
sciCAR: Single-cell combinatorial indexing of chromatin accessibility and mRNA
SCITO-seq: Single-cell combinatorial indexed cytometry sequencing
SC-MEB: Spatial clustering using the hidden Markov random field based on empirical bayes
scMT-seq: Single-cell methylome and transcriptome sequencing
scM&T-seq: Single-cell genome-wide methylome and transcriptome sequencing
scNMT-seq: Single-cell nucleosome, methylation and transcription sequencing
scRNA-seq: Single-cell RNA sequencing
scTHS-seq: Single-cell transposome hypersensitive site sequencing
scTrio-seq: Single-cell triple omics sequencing
SEDAL: Sequencing with error-reduction by dynamic annealing and ligation
seqFISH: Sequential FISH
SIDR: Simultaneous isolation of genomic DNA and total RNA
smFISH: Single-molecule fluorescence in situ hybridization
smHCR: Single-molecule hybridization chain reaction
SMI: Spatial molecular imaging
SM-omics: Spatial multi-omics
SNARE-seq: Single-nucleus chromatin accessibility and mRNA expression sequencing
snDrop-seq: Single-nucleus droplet-based sequencing
snoRNA: Small nucleolar RNA
SNVs: Single nucleotide variants
spatial-CITE-seq: Spatial co-indexing of transcriptomes and epitopes
SARS-CoV2: Severe acute respiratory syndrome coronavirus 2
SPOTS: Spatial protein and transcriptome sequencing
ST: Spatial transcriptomics
STARmap: Spatially-resolved transcript amplicon readout mapping
Stereo-seq: Spatial enhanced resolution omics sequencing
STRS: Spatial total RNA sequencing
SVCA: Spatial variance component analysis
TAMs: Tumor-associated macrophages
TIVA: Transcriptome in vivo analysis
TLS: Tertiary lymphoid structures
TME: Tumor microenvironment
tRNA: Transfer RNA
UMI: Unique molecular identifier
xDBiT: Multiplexed deterministic barcoding in tissue
yPAP: Yeast poly(A) polymerase
3D: Three-dimensional
4sU: 4-Thio-uridine
5-EU: 5-Ethynyl-uridine
